# Correlation between Plasma Interleukin-3, the *α*/*β* Globin Ratio, and Globin mRNA Stability

**DOI:** 10.1155/2014/640203

**Published:** 2014-11-18

**Authors:** S. Rouhi Dehnabeh, R. Mahdian, S. Ajdary, E. Mostafavi, S. Khatami

**Affiliations:** ^1^Biochemistry Department, Pasteur Institute of Iran, Pasteur Street, No. 69, Tehran 1316943551, Iran; ^2^Molecular Medicine Department, Pasteur Institute of Iran, Pasteur Street, No. 69, Tehran 1316943551, Iran; ^3^Immunology Department, Pasteur Institute of Iran, Pasteur Street, No. 69, Tehran 1316943551, Iran; ^4^Department of Epidemiology, Pasteur Institute of Iran, Pasteur Street, No. 69, Tehran 1316943551, Iran

## Abstract

*Background*. Globin chain synthesis (GCS) analysis is used in the diagnosis of thalassemia. However, the wide reference range limits its use as a decisive diagnostic tool. It has been shown that *α* and *β* *globin mRNA* increase through stimulation of cells by interleukin-3 (IL-3). Therefore, this study investigates the relationship between plasma IL-3 and the *β*/*α* *globin* ratio.* Methods*. Blood samples were collected from 32 healthy participants on two occasions one month apart. GCS analysis, real-time PCR, and ELISA tests were conducted to determine the *β*/*α* *globin* ratio, *globin mRNA* expression and stability rate, and IL-3 levels.* Results*. On the basis of IL-3 levels, the participants were divided in two groups. One group included participants who showed a significant increase in IL-3 as indicated by a significant rise in mean values of *α*, *β*, and *γ* *globin mRNA*, *α* and *β* *globin*, RBC, and hemoglobin. The other group included participants who showed no difference in IL-3 levels with no significant variations in the above-mentioned parameters.* Conclusion*. The results of this study indicate that IL-3 has an equivalent positive effect on *α* and *β* *globin* chain synthesis. Therefore, IL-3 levels do not explain the wide reference range of the *α*/*β* *globin* ratio.

## 1. Introduction

Thalassemia is one of the most common genetic diseases in Iran. In an attempt to limit the emergence of new cases of major *β*-thalassemia, health authorities are developing a national program to accurately diagnose different types of thalassemia. With more than 200 genetic mutations, thalassemia has different clinical manifestations ranging from no symptoms to severe disease. Currently, despite the fact that* DNA* analysis provides useful information on thalassemia, it cannot be used alone as a decisive diagnostic tool. This is because the disease involves unknown deletions and mutations, mutations in gene regulation sites, and mutations in genes generating trans-elements for globin gene expression (nonglobin gene related thalassemia). In some cases, globin chain synthesis is required for diagnosis because it enables monitoring of gene expression at different levels, including transcription (*mRNA* production), generation of stable* mRNA*, and translation on ribosomes [1]. In the globin chain synthesis carried out to determine the *α*/*β* *globin* ratio, all intermediate stages of globin generation (from no generation of* mRNA* or unstable* mRNA* to no translation on ribosomes) are controlled. Therefore, the results of this test indicate the final efficiency of globin genes and are very valuable for diagnosis. However, the wide reference range [2, 3], a consequence of the greater influence of biological changes compared with methodological changes [2, 4], limits the use of this method as a decisive and final diagnostic tool. To address this, it is necessary to study the effects of biological substances on globin chain synthesis test results.

Previous research has shown that interleukin-3 (IL-3) can alter globin chain synthesis, which in turn affects translation levels. In this way, IL-3 generates hemoglobin F through its stimulating effect on *α* *globin* and *γ* *globin* chain synthesis [5].

IL-3 is lymphocytes, epithelial cells, and astrocytes secreted cytokine. The IL-3 molecule has a glycoprotein structure and is generated as a result of antigenic or mitogenic stimulation. It affects the reproduction and differentiation of blood cells and other cells through special receptors [6]. IL-3 can promote erythropoiesis by activating the Ras pathway, resulting in apoptosis control and a Jak2/stat5 cascade, and by stimulating* DNA* synthesis [7].

The hemoglobin molecule has a complex structure, and the components contributing to the molecular structure are coordinated through complex mechanisms. However, coordinated generation of the chains contributing to the structure of hemoglobin depends on other factors, including blood iron level, ferritin concentration, transferrin receptors, cytokine concentration, and heme concentration [8]. In addition, the generation of protein subunits contributing to the hemoglobin structure is coordinated so the generation rate of *α* chains almost approaches that of non-*α* chains. This process prevents cell damage from increases in levels of one type of chain but is disrupted in thalassemia.

The present study aims to investigate the specific effect of IL-3 on *α* *globin mRNA* and *β* *globin mRNA* stability, *α* *globin* and *β* *globin* production, and the range of the *α*/*β* *globin* ratio, assuming that antigenic and mitogenic stimulation results in different concentrations of IL-3 in the blood at different times (affecting the stability of the exclusive * mRNA globin* as well as the generation of *α* and *β* chains).

## 2. Material and Methods

Blood samples were collected from 32 healthy participants, on two occasions one month apart. The participants were divided in two groups based on IL-3 levels. Group 1 included participants who showed a significant increase in IL-3 levels over the month, and Group 2 included participants who showed no change or decrease in IL-3 levels.

Inclusion criteria based on blood sample analysis were as follows: MCV > 80 fL, MCH > 27 pg, normal levels of total iron, TIBC, and ferritin, and normal hemoglobin electrophoresis. All participants provided written, informed consent prior to taking part in the study.

Complete blood count (CBC) tests were carried out using a Sysmex KX-1000 apparatus and hemoglobin electrophoresis was performed on acetate cellulose. Total iron concentration and TIBC and ferritin levels were measured using the colorimetric method (Darman Kav Co. kit) and the ELISA method (Padtan-Teb Company kit), respectively.

At the start of the study and after one month, globin chain synthesis, real-time PCR, and ELISA tests were conducted to determine the*α*/ *β* *globin *ratio (globin chain generation), * globin mRNA* expression and stability rate, and the level of IL-3, respectively.

The Weatherall and Clegg method [9] was used for globin chain synthesis to determine the *α*/*β* *globin* ratio. Globin chain analyses were done using Mono-S ion exchange columns and a high-performance liquid chromatography (HPLC) device [2].

The IL-3 concentration was measured using the ELISA method (R&D Company kit Lot. no. 765250.1).

Real-time PCR was used to investigate the expression and stability of *α* *globin mRNA*, *β* *globin mRNA*,  and *γ* *globin mRNA*, [10–12]. First, the * mRNA *was purified and * cDNA* was produced, and real-time PCR was performed. Standard graphs were constructed using a serial dilution of * cDNA* samples from healthy people to determine the efficiency of PCR for each gene, including the *α* *globin*, *β* *globin*, and *γ* *globin* genes, and the * GAPDH *reference gene.

Tri Reagent (Sigma, DB) was used to purify * mRNA*, which was kept in water containing diethyl pyrocarbonate (DEPC). To verify the quality of the * mRNA*, the optical absorption was measured at 260 and 280 nm wavelengths using a Nanodrop spectrometer (Implen, Munich, Germany). The mean optical absorption of the purified samples was 2.02. A Qiagen kit (cat. number 205311) was used to make * cDNA*. The real-time PCR test was performed using an ABI 7300 Sequence Detection System (Applied Biosystems, Foster City, CA, USA). The oligonucleotide primers for *α* , *β* , and *γ* *globin *genes and the * GAPDH* reference gene were designed with Primer Express software Ver. 3 (Applied Biosystems, Foster City, CA). The specificity of the primer sequences was confirmed on a search of the NCBI/BLAST database. The characteristics of the primers are shown in Table 1.

Following the determination of the best concentration of * cDNA*, to avoid the generation of dimer primers, a real-time PCR test was conducted on special plates to ensure the optimal reaction and concentration of primers. For each reaction, a solution with a volume of 25 mL was prepared. The contents of the solution were as follows: Power SYBR Green Master Mix (Applied Biosystems, UK), 12.5 mL; *α*, *β*, and *γ* primers, 3 pmol; * GAPDH* primer, 5 pmol; water, 6.5–7 mL; and * cDNA*, 25 ng. The test was conducted for all four genes in two simultaneous series and the mean Ct was computed for each gene.

The following schedule was used for the real-time PCR test.

The first cycle was carried out at 95°C for 10 min, to achieve primary separation of the expression pattern * cDNA*. Next, two thermal schedules were repeated for 40 cycles: 95°C for 15 s and 60°C for 1 min.

For every complete reproduction stage, a separation step was carried out to analyze the melting curve: 95°C for 15 s, 60°C for 30 s, and 90°C for 15 s.

To determine the efficiency of reproduction [11, 12] of *α*, *β*, and *γ* *globin *genes and the* GAPDH* reference gene, a serial dilution of the * cDNA* sample from one of the study participants was prepared and a real-time PCR test was carried out separately, using different primers and different concentrations. Finally, a standard graph was drawn for each of the four gene sections. The efficiency of PCR for all four genes was calculated through the determination of the standard graph slope and was derived from the following equation:

(1) $$\begin{array}{c} \text{Efficiency}=\left[10^{\left(-1/\text{Slope}\right)}\right]-1. \end{array}$$

**Figure 1:**
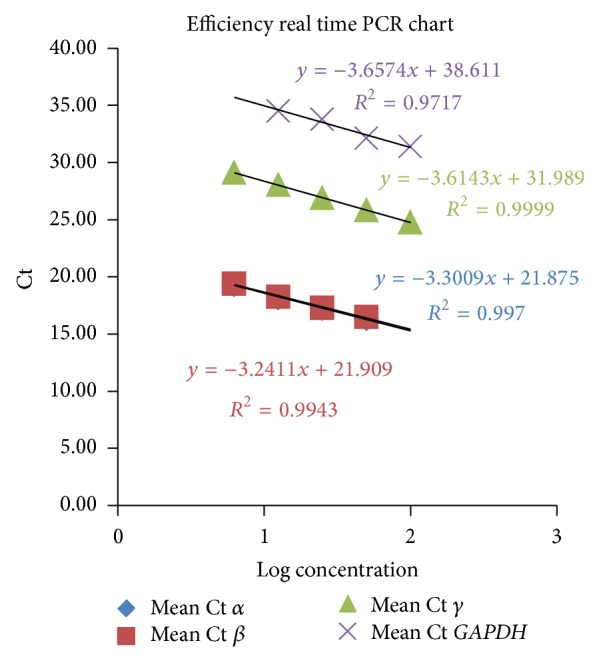
The standard curves of the real-time PCR assay.

**Figure 2:**
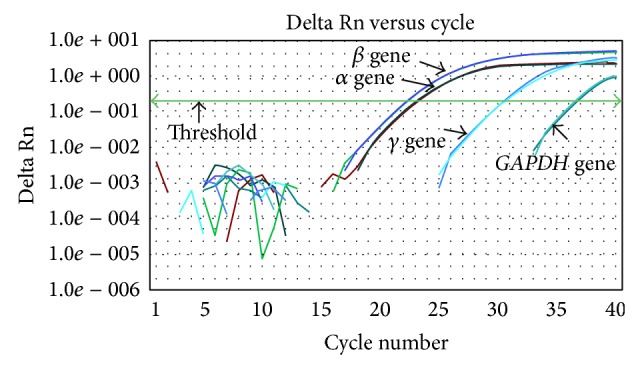
Delta Rn versus cycle for different genes.

**Figure 3:**
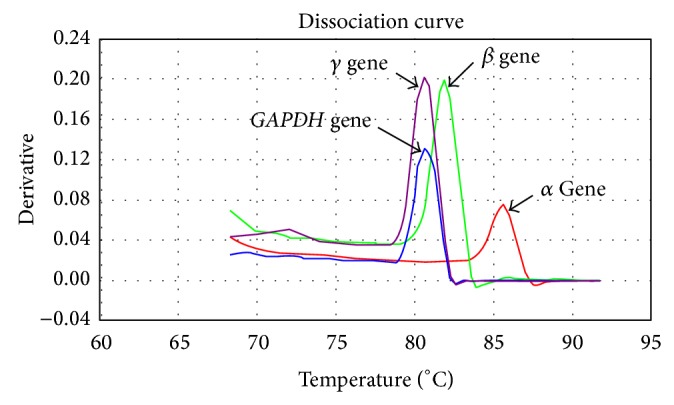
Dissociation curve for different genes.

When the efficiency was sufficient to allow the use of the 2^−ΔΔCt^ method (Table 2 and Figure 1), the real-time PCR test was performed on *α*, *β*, and *γ* *globin* genes in the samples from the participants and the * GAPDH* reference gene. When reproduction was complete, a graph was drawn for each PCR reaction, based on the Ct (Figures 2 and 3). A ΔCt index was calculated for standard and study samples by deducing the mean Ct of the target genes and of the reference gene, resulting in the ΔΔCt factor, which is derived from the following equation:

(2) ΔΔCt=mCt  αtest  Sample−mCT  GAPDHtest  Sample −mCT  α(normal  Sample)  i−mCT  GAPDHnormal  Sample.

Finally, the expression rate of the *α*, *β*, and *γ* *globin* was determined using the 2^−ΔΔCt^ formula.

Based on the research objectives and assumptions, the results were analyzed, compiled, and interpreted using SPSS ver. 16 (SPSS, Chicago, IL, USA).

## 3. Results

On the basis of IL-3 levels, the participants were divided in two groups. One group included 15 participants who showed a significant increase in IL-3 after one month and the other group included 17 participants who showed no difference or decrease in IL-3 levels.

Table 3 shows the mean ± standard deviation of the results and demonstrates that all participants had normal MCV and MCH values and no evidence of iron deficiency.

The Kolmogorov-Smirnov test was used to test the dispersion of the studied variables, and the mean values of the following variables were statistically analyzed and compared: *α* *globin mRNA*, *β* *globin mRNA*, *γ* *globin mRNA*, *α* *globin*, *β* *globin*, *γ* *globin*, *α*/*β* *globin ratio*, RBC, hemoglobin, IL-3, ferritin, and the reticulocyte count.  Table 4 presents a summary of the results. The variables followed a normal distribution. Therefore, a paired *t*-test was used to compare the variables between groups. In Group 1 (participants with increased IL-3), the mean values of *α* *globin mRNA*, *β* *globin mRNA*, *γ* *globin mRNA*, *α* *globin*, *β* *globin*, RBC, hemoglobin, and IL-3 all increased over the month of the study. The increase was significant for *α* *globin mRNA*, *β* *globin mRNA*,  and  *γ* *globin mRNA* at a significance level of 90% and for hemoglobin, RBC, and IL-3 at a significance level of 95%. In Group 2, the mean concentration of IL-3 showed a decrease after one month, which was significant at a level of 95%. Among the parameters studied using the paired *t*-test, none showed a significant difference between the two test steps with a resampling interval of one month. In the present study of healthy participants, the *α*/*β* *globin* ratio range was 0.74–1.31, which agrees well with the findings of recent studies [2, 3].

## 4. Discussion

Many studies have demonstrated that transcriptional control mechanisms regulate gene expression, and recently, research has confirmed the importance of posttranscriptional mechanisms in the regulation of eukaryotic gene expression [1]. Increased* mRNA* reading by ribosomes [13], increased protection against proteins produced from degradation mechanisms [14], and increased stability of* mRNA* are possible mechanisms underlying changes in translation levels. Previous research has shown that, in addition to increases in transferrin (CD71) receptors, *α* *globin mRNA* and *β* *globin mRNA* increase through stimulation of cells by IL-3. This increase is attributed to the stabilization of *α* *globin mRNA* and *β* *globin mRNA* molecules [5].

The decrease in ferritin levels in the group 1 with increased IL-3 in the present study could be attributed to increased CD71 receptors as well as increased IL-3 (CDw123) receptors. The decreased ferritin levels could be attributed to the requirement for iron to produce heme molecules. The presence of CD71 receptors and CDw123 receptors on reticulocytes has been confirmed [5]. It has also been shown that the number of CD71 receptors increases as IL-3 concentration increases [5]. This may occur because an increase in * globin mRNA* will result in an increase in translation levels. Further, since the generation of hemoglobin molecules requires a heme component, the number of CD71 receptors will increase to enable the generation of heme molecules.

The complex process of hemoglobin generation is dependent on many factors, including the blood iron level, ferritin concentration, transferrin receptors, cytokine (IL-3 and IL-9) concentrations, heme concentration, the hematopoietic GTPase RhoH required for adjusting the signal effects of IL-3 [15], and the presence or absence of CD133 on the surface of erythroid cells. These factors cause adult cells to react to IL-3 to more strongly activate erythropoiesis [16]. It is difficult to explain why the reference range of the *α*/*β* *globin* ratio is so wide and future comprehensive research is required to answer this question. Recent studies have shown that the expression of IL-3 receptors, as well as signal transducers and activators of transcription (STAT) activity, is adjusted under the influence of GTPase RhoH, which in turn results in the adjustment of IL-3 signals [15].

The results of this study on the *α*/*β* *globin* ratio imply that because IL-3 has an equivalent positive effect on the generation of *α* *globin* and *β* *globin*, it is not likely to be the biological factor underlying the wide range of *α*/*β* *globin* ratios in globin chain synthesis tests.

## Figures and Tables

**Table 1:** The primers' sequences.

(γ)	Seq 5′→3′	Tm	GC%	L	L
F	CAAGGTGAATGTGGAAGATGCTG	60.5	48	23	115 bp
R	GATGGCAGAGGCAGAGGACAG	60.9	62	21	

(β)	Seq 5′→3′	Tm	GC%	L	L

F	CGTGGATGAAGTTGGTGGTGAG	61.3	55	22	112 bp
R	GCCCATAACAGCATCAGGAGTG	60.5	55	22	

(α)	Seq 5′→3′	Tm	GC%	L	L

F	GCACAAGCTTCGGGTGGAC	60.2	63	19	157 bp
R	GGTATTTGGAGGTCAGCACGGT	61.1	55	22	

**Table 2:** Determination of the real-time PCR efficiencies.

	Concen.	Log	Mean Ct	Ct1	Ct2	Ct3	Slope	Effic. %
*α*	50	1.69897	16.32	16.32	16.31	16.33	−3.30089	101
	25	1.39794	17.22	17.21	17.23	17.22		
	12.5	1.09691	18.17667	18.22	18.15	18.16		
	6.25	0.79588	19.31333	19.27	19.31	19.36		

*β*	50	1.69897	16.48667	16.46	16.47	16.53	−3.24109	103
	25	1.39794	17.29	17.28	17.3	17.29		
	12.5	1.09691	18.27667	18.32	18.17	18.34		
	6.25	0.79588	19.41	19.45	19.36	19.42		

*γ*	100	2	24.76667	24.54	24.92	24.84	−3.61426	89
	50	1.69897	25.85	25.82	25.95	25.78		
	25	1.39794	26.91	26.94	26.88	26.91		
	12.5	1.09691	28.04333	28.05	28.05	28.03		
	6.25	0.79588	29.11	29.02	29.14	29.17		

*GAPDH *	100	2	31.395	31.3		31.49	−3.65744	88
	50	1.69897	32.11	32.06		32.16		
	25	1.39794	33.775	33.52	34.03			
	12.5	1.09691	34.51		34.85	34.17		

**Table 3:** Hematological and biochemical data.

Variables	Group 1 Mean ± 2 SD	Group 2 Mean ± 2 SD
WBC (×10^3^/L)	6.7 ± 2.0	6.5 ± 2.2
RBC (×10^12^/L)	4.89 ± 0.80	4.84 ± 0.80
Hb (g/dL)	14.0 ± 2.0	13.8 ± 2.6
Hct (%)	41.7 ± 5.4	41.3 ± 6.8
MCV (fL)	85.4 ± 5.2	85.5 ± 5.0
MCH (pg)	28.7 ± 1.8	28.5 ± 1.4
MCHC (g/dL)	33.6 ± 1.2	33.0 ± 2.4
RDW	12.0 ± 0.7	12.4 ± 1.8
Reticulocytes (%)	0.9 ± 0.8	0.8 ± 0.6
Hb A (%)	97.1 ± 0.6	97.2 ± 0.5
Hb A_2_ (%)	2.3 ± 0.6	2.4 ± 0.4
Hb F (%)	0.5 ± 0.4	0.4 ± 0.3
Total iron (ug/d)	86 ± 33	99 ± 57
TIBC (ug/dL)	339 ± 88	328 ± 138
Ferritin (ng/dL)	46 ± 102	56 ± 132
Number of total cases	15	17

**Table 4:** Summary of the results.

Parameter	Step 1 of sampling in Group 1 (*n* = 15) Mean (SD)	Step 2 of sampling in Group 1 (*n* = 15) Mean (SD)	Step 1 of sampling in Group 2 (*n* = 17) Mean (SD)	Step 2 of sampling in Group 2 (*n* = 17) Mean (SD)
*α* *globin mRNA *	1.00 (0.0)	3.99 (5.10)	1.00 (0.0)	1.24 (1.16)
*β* *globin mRNA *	1.00 (0.0)	3.73 (5.14)	1.00 (0.0)	1.23 (0.97)
*γ* *globin mRNA *	1.00 (0.0)	2.79 (3.08)	1.00 (0.0)	1.12 (0.84)
*α* *globin *	100.00 (0.0)	129.87 (17.63)	100.00 (0.0)	100.11 (41.45)
*β* *globin *	100.00 (0.0)	134.93 (72.46)	100.00 (0.0)	102.29 (46.56)
*γ* *globin *	100.00 (0.0)	105.07 (28.49)	100.00 (0.0)	98 (41.65)
*α*/*β* *globin* ratio	1.083 (0.118)	1.058 (0.129)	0.99 (0.14)	0.96 (0.13)
RBC	4.69 (0.44)	4.89 (0.36)	4.83 (0.46)	4.83 (0.42)
Hemoglobin	13.58 (1.05)	14.01 (0.96)	13.95 (1.40)	13.78 (1.34)
IL-3	25.67 (28.76)	67.07 (75.60)	46.64 (50.95)	29.35 (23.28)
Ferritin	52.87 (59.81)	44.80 (51.51)	57.00 (56.34)	56.12 (66.37)
Concentrated reticulocyte	2.13 (0.80)	2.18 (0.75)	1.87 (0.52)	1.78 (0.52)
